# Abdominal Morphologic Changes in MRI during Gastric Balloon Therapy

**DOI:** 10.1159/000526444

**Published:** 2022-08-16

**Authors:** Rebecca Keßler, Anne Glitsch, Björn Hübner, Simone Gärtner, Antje Steveling, Maciej Patrzyk, Wolfram Keßler

**Affiliations:** ^a^Department of Diagnostic Radiology and Neuroradiology, University Medical Center Greifswald, Greifswald, Germany; ^b^Department of General, Visceral, Thoracic and Vascular Surgery, University Medical Center Greifswald, Greifswald, Germany; ^c^Department of Internal Medicine A, University Medical Center Greifswald, Greifswald, Germany

**Keywords:** Gastric balloon, Obesity, Liver volume, Sarcopenic adiposity, Fatty tissue

## Abstract

**Introduction:**

Adiposity and excessive weight are on the rise in western industrialized countries. In cases where conservative measures fail and surgical interventions are not (yet) desired, gastric balloon therapy has proven to be a safe and reversible endoscopic method.

**Methods:**

Aside from weight progression under gastric balloon therapy and by using MRI, our research paper describes the behavior of different abdominal body fat compartments at the beginning and at the end of the gastric balloon therapy. Additionally, the volume of the left liver lobe as well as the fill volume and performance of the gastric balloon were analyzed over the duration of treatment. For assessing potential impacts of weight reduction on the muscle mass, we determined the area of the m. psoas on a comparable cross-sectional area at the beginning and at the end of the therapy.

**Results:**

We were able to verify a significant reduction of the layer of subcutaneous fat, adipose capsule of the kidney, and intra-abdominal fatty tissue during the therapy. The volume of the left liver lobe was shrinking in addition to a muscle loss during the balloon therapy. The volume of the gastric balloon remained stable (not hyperinflation). There were variable gas bubbles in the gastric balloon.

**Conclusion:**

The gastric balloon is a temporary and successful option for weight reduction by reducing body fat, liver volume, but also muscle mass.

## Introduction

Excessive weight and obesity are widespread diseases with an increasing prevalence [1, 2, 3]. The initial therapy approach is a combination of dietary measures and increased physical activity [4]. Nevertheless, in many cases, morbid adiposity cannot be managed with conservative therapy concepts [4, 5]. The gastric balloon insertion is a reversible endoscopic therapy option [6, 7, 8, 9]. It can be performed either within the context of weight reduction [10] or in preparation of a future bariatric surgery [11, 12]. Specific effects of this therapy on the fatty tissue distribution and liver volume must be assumed but are rarely described. MRI scans seem to be suited for documenting the fatty tissue reduction regarding the compartment changes [13, 14]. MRI-based measurement of the m. psoas as sentinel muscle can also provide an objective assessment of a change in the muscle mass [15]. A predictively unfavorable impact seems to be a combination of high body fat content and low muscle mass, the so-called sarcopenic adiposity [16]. A loss of muscle mass must be avoided independent of the therapy approach.

Since 2017, a spontaneous hyperinflation of liquid-filled gastric balloons has been discussed by the US Food and Drug Administration (FDA) [17]. Changes in the volume of gastric balloons can be measured by using MRI.

Our present study assesses weight reduction under gastric balloon therapy, particularly regarding its effect on different fatty tissue compartments as well as on the volume of the left liver lobe. By using MRI, the undesirable effect on the patients' muscle mass is also assessed, as is the performance of the gastric balloon over the duration of treatment.

## Methods

### Data Collection

The data were collected from 2012 to 2018. Admitted to the study were patients, where according to the applicable German guidelines [18] at that time, bariatric therapy was indicated (BMI over 40 kg/m^2^ or BMI over 35 kg/m^2^ with comorbidities), who had previously been offered the gastric balloon therapy as reversible procedure or this procedure had been desired by the patients. The study was approved by the local University Medical Center Ethics Commission (Ethikkommission an der Universitätsmedizin Greifswald, Institut für Pharmakologie, Felix-Hausdorf-Straße 3, 17487 Greifswald, Germany) under the registration number BB24/12. Written informed consent was obtained from all individual participants included in the study. The study included 10 female and 4 male patients with successfully implanted gastric balloons. One female patient noticed the passing of the gastric balloon via naturalis during the removal preparation. This day was regarded as removal day. The balloons of 2 female patients had to be removed ahead of the schedule due to symptoms (1× heartburn, 1× excruciating epigastric pain). These patients were considered as dropouts.

### Gastric Balloon Insertion and Removal

The insertion and removal of the gastric balloon (BIB^TM^ System Intragastric Balloon; Apollo Endosurgery, Austin, Texas, USA) were carried out in analgosedation (Diprivan ± Midazolam) in compliance with the only just, in 2015, published guidelines on sedation in endoscopy. During the calibration (filling) of the gastric balloon, care was taken to ensure a smooth stomach passage with the endoscope (filling volume 708.3 ± 23.7 mL (mean ± SEM), minimum 500 mL, maximum 850 mL). The patients were administered with PPI medication (40 mg Pantoprazole 1× daily p.o.) over the duration of treatment.

### MRI Examination

MRI scans were performed using a 1.5-Tesla scanner (Magnetom Aera; Siemens Healthcare GmbH, Erlangen, Germany). Trained technicians performed all examinations in a standardized way. Images were acquired in a supine position using a combination of a spine and body phased-array coil covering the upper abdomen. The MRI protocol was identical for all participants and included three scans of the upper abdomen. The MRI protocol consisted of T2-weighted HASTE (Half-Fourier acquisition single-shot turbo spin echo) sequences in coronal, sagittal, and axial plane. Imaging parameters for this sequence in the coronal and sagittal plane were as follows: TR/TE: 1,300/91 ms; flip angle 180°; turbo factor 256; bandwidth 698 Hz/px; imaging matrix: 256; field of view 400 mm, parallel acquisition (Grappa) with an acceleration factor of 2 and 42 reference lines, slice thickness 6.0 mm, 30 slices with a distance factor of 20%. The images were obtained in multi-breath-hold acquisition with a total acquisition time of 49 s. The imaging parameters for the axial plane differed as follows: turbo factor 198, field of view 423 mm, all further imaging parameters were equal to the coronal/sagittal plane. The images were obtained in multiple breath-hold periods with a total acquisition time of 47 s.

All patients were examined twice with the initial scan performed shortly after the balloon insertion and the follow-up scan directly preceding the balloon removal. MRI image reading for all scans was performed by using OsiriX Lite for Mac (Version 10.0; Pixmeo SARL, Bernex, Switzerland), an image processing software dedicated to DICOM images. All acquired images were evaluated by one reviewer (10 years of experience in diagnostic radiology).

On initial scans and follow-up imaging, the gastric balloon was assessed for its correct location in the gastric fundus and partially corpus, its volume and special filling characteristics (gas bubbles). The balloon volume was recorded as an average volume from volume measurements on all three planes by using circular regions of interest (ROIs) manually placed on each slice depicting the balloon. For each examination, the volume of the left liver lobe was determined as an average volume from volume measurements on all three planes by using polygonal ROIs manually placed on all slices depicting the left liver parenchyma.

Additional image evaluation focused on the assessment of the abdominal adipose tissue. The fatty tissue was measured in three locations to cover all compartments of the abdominal fat (subcutaneous, visceral, retroperitoneal): subcutaneous fat on the anterior median abdominal wall 5 cm caudal to the xiphoid process on sagittal plane images, visceral fat of the mesenteric root as distance between the anterior aortic wall and the posterior pancreatic organ capsule on axial plane images, and retroperitoneal fat of the right renal capsule in the middle third of the kidney measured at the lateral circumference on axial plane images. Additionally, we calculated the skeletal muscle mass using the psoas muscle as a sentinel and measuring its area on axial plane images at the level of the third lumbar vertebra on both sides by means of a manually selected polygonal ROI.

### Statistics

**Fig. 1:**
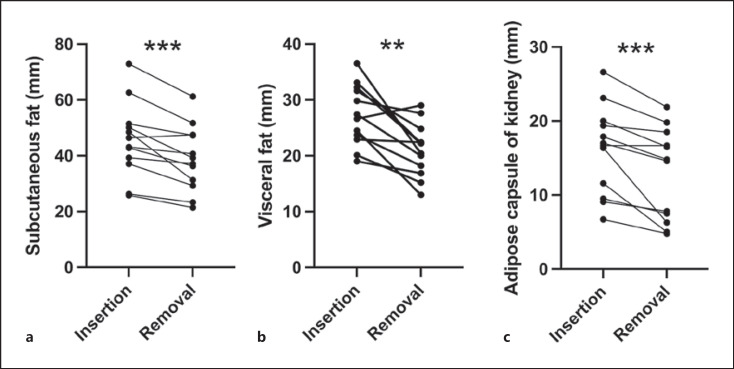
Changes of the fatty tissue per patient over the course of the gastric balloon therapy. **a** The subcutaneous fat decreases from 45.53 ± 3.88 mm to 38.86 ± 3.39 mm (mean ± SEM) (***p = 0.001, *n* = 12). **b** The visceral fat decreases from 27.28 ± 1.58 mm to 21.00 ± 1.38 mm (mean ± SEM) (**p = 0.0034, *n* = 12, pairing not significant). **c** The adipose capsule of kidney decreases from 16.17 ± 1.72 mm to 12.85 ± 1.79 mm (mean ± SEM) (***p = 0.001, *n* = 12).

A Wilcoxon matched-pairs signed-ranks tests was performed using GraphPad Prism (Version 8.0.0 for Windows; GraphPad Software, San Diego, CA, USA, www.graphpad.com). When data are shown as significantly different, the *p* value is two-tailed, and the pairing was effective (Fig. 1b, pairing was not significantly effective).

## Results

### Patient Characteristics and Weight Progression

Twelve patients (8 female and 4 male) between 21 and 59 years of age (mean ± SEM: 37.36 ± 3.63 years) were examined with MRI short after the gastric balloon insertion and before removal. The duration of gastric balloon treatment was between 25 and 43 weeks (30.1 ± 1.6 weeks, mean ± SEM), initially showing a BMI between 39.1 kg kg/m^2^ and 67 kg/m^2^ (52.51 ± 2.35 kg/m^2^, mean ± SEM). BMI at removal was between 36.84 kg/m^2^ and 61.05 kg/m^2^ (46.92 ± 1.90 kg/m^2^, mean ± SEM). One patient showed no BMI reduction. All patients' BMI reduction through balloon therapy presented a statistically high significant impact (***p = 0.0005, 52.51 ± 2.35 kg/m^2^ vs. 46.92 ± 1.90 kg/m^2^, mean ± SEM, *n* = 12).

### Changes of Fat Distribution in the Upper Abdomen

For assessing the fat distribution over the course of the gastric balloon therapy, we chose locations that were likely to facilitate reproducible measurement results due to the fatty tissue's low mobility when changing the position. Viewing the subcutaneous fatty tissue 5 cm below the xiphoid in the midline (Fig. 1a), we noticed a significant reduction (45.53 ± 3.88 mm vs. 38.86 ± 3.39 mm, mean ± SEM, ***p = 0.001, *n* = 12) of the fatty tissue layer. Regarding the visceral fatty tissue, we viewed the thickness in millimeters from the aorta to the corpus pancreatis. This is where we noticed a highly significant reduction over the course of the gastric balloon therapy (27.28 ± 1.58 mm vs. 21.00 ± 1.38 mm, mean ± SEM, **p = 0.0034, *n* = 12, Fig. 1b, pairing not significantly effective). The examination of the retroperitoneal fat compartment (thickness in millimeters, lateral of the right middle third of the kidney) also revealed a significant reduction over the course of the treatment (16.17 ± 1.72 mm vs. 12.85 ± 1.79 mm, mean ± SEM, **p = 0.001, *n* = 12, Fig. 1c).

### Volume Change of the Left Liver Lobe and Psoas Area

**Fig. 2:**
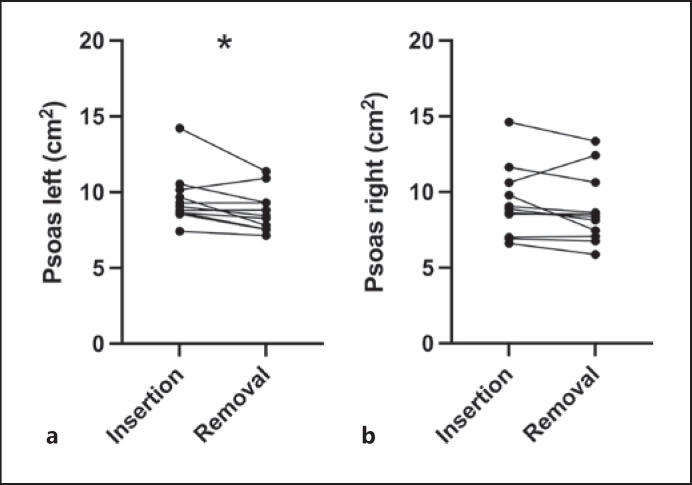
Depiction of separate sides of the psoas sectional area per patient over the course of the gastric balloon therapy. **a** The left psoas sectional area decreases over the course of the gastric balloon therapy from 9.55 ± 0.53 cm^2^ to 8.78 ± 0.42 cm^2^ (mean ± SEM, *p = 0.0322, *n* = 11). **b** The right psoas sectional area covers an area of 9.31 ± 0.71 cm^2^ (mean ± SEM) at the time of insertion and does not decrease significantly over the course of the therapy (sectional area at removal: 8.85 ± 0.71 cm^2^, mean ± SEM, *p* = 0.083, *n* = 11).

**Fig. 3:**
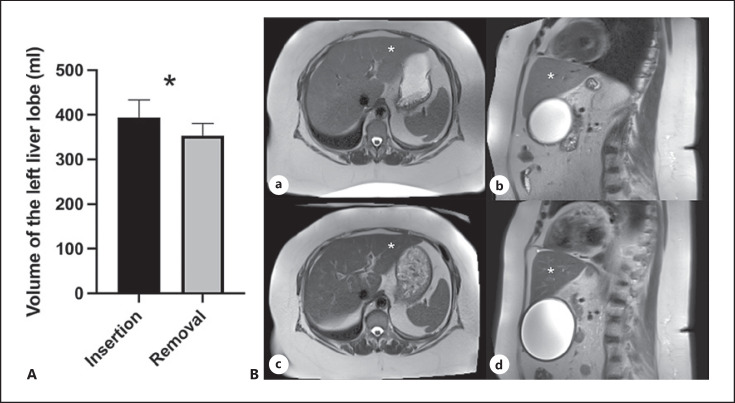
Volume changes of the left liver lobe. **A** The volume of the left liver lobe decreases over the course of the therapy from 394.0 ± 39.27 mL to 353.4 ± 27.68 mL (mean ± SEM) (*p = 0.0493, *n* = 12). **B** T2-weighted images of a 60-year-old female patient in axial and sagittal plane (**a, b**, initial scan; **c, d**, follow-up scan) illustrating a decrease in size of the left liver lobe (*) over a period of approximately 9 months. Note the decrease in liver signal intensity in image **c, d** as a result of declining fatty liver.

The cross-sectional area of m. psoas at the level and to the right side of the 3rd lumbar vertebra comprised 9.31 ± 0.71 cm^2^ (mean ± SEM) at the time of insertion and 8.85 ± 0.71 cm^2^ (mean ± SEM, see Fig. 2b) at the time of the gastric balloon removal, thus showing no significant change (*p* = 0.083, *n* = 11). However, the left side showed a significant reduction of the muscle's sectional area at the level of the third lumbar vertebra (9.55 ± 0.53 cm^2^ vs. 8.78 ± 0.42 cm^2^, mean ± SEM, *p = 0.0322, *n* = 11, see Fig. 2a). Consequently, we have to assume a muscle mass loss under the therapy. The left liver lobe's volume showed a significant reduction from 394 ± 39.27 mL to 353.4 ± 27.68 mL (mean ± SEM) over the course of the therapy (*p = 0.0493, *n* = 12, see Fig. 3A, B).

### Assessment of the Gastric Balloon

**Fig. 4:**
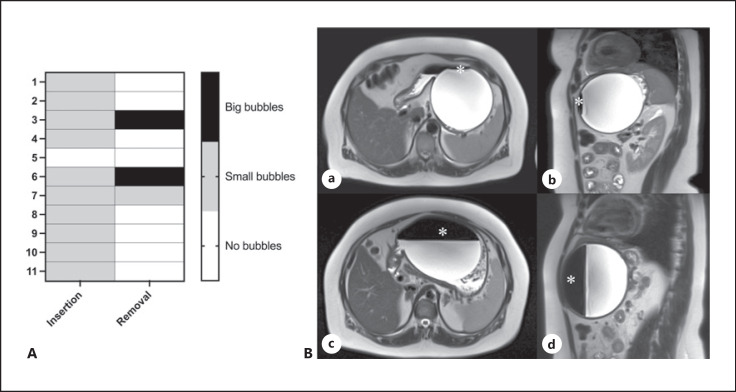
Volume changes of bubbles in the gastric balloon. **A** The detectable air bubbles showed dynamics at the time of the insertion. In 8 of 11 cases, the air bubbles had disappeared by the time of removal, in 2 cases, the bubbles had increased (patient 3 and 6) and 2 cases showed a stable situation (patient 5 and patient 7 without bubbles). One balloon was passed via naturalis and was thus excluded. **B** T2-weighted images of a 48-year-old female patient in axial and sagittal plane (**a, b**, initial scan; **c, d**, follow-up scan) depicting an enlargement of the gas bubble (*) in the gastric balloon over a period of approximately 6 months. The balloon volume increased from 730 cm^3^ to 1,113 cm^3^.

The gastric balloon's measured fill volume was 696.2 ± 20.8 mL (mean ± SEM) at the time of insertion. At the time of removal, we were able to verify a fill volume of 732.9 ± 49.85 mL (mean ± SEM). Thus, no significant change in the measured fill volume could be detected over the duration of treatment (*p* = 0.97, *n* = 11). By assessing the balloon, changes regarding gas bubbles in the balloon became apparent. While initially small gas bubbles could be verified in 10 of 11 balloons, at the time of removal, 8 of 11 balloons no longer showed any gas bubbles at all. Two balloons displayed an increase in bubble formation (see Fig. 4A, B) and one balloon showed a stable situation with small gas bubbles within the lumen (see Fig. 4A). One balloon was passed via naturalis and could no longer be verified in the MRI.

## Discussion

Morbid adiposity and thus associated comorbidities have become widespread diseases [1, 2, 3]. The endoscopic gastric balloon insertion was applied by us in individual cases from 2012 to 2018 as a reversible endoscopic method of therapy [6, 7, 8]. It can be performed within the context of weight reduction [10] or in preparation of bariatric surgery at a later point in time [11, 12, 19] (“multi-stage approach”). The current German guidelines on adiposity surgery recommend a gastric balloon only in the context of case-by-case decisions or if conservative measures fail in adipose patients and they oppose surgical therapy [4]. In 2017, the FDA documented and published 33 therapy-associated cases of death [17]. Stavrou et al. [9] discussed investigator-related technical problems by the use of gastric balloons. In 2020, the FDA recommended to monitor patients closely during the entire duration of treatment with liquid-filled intragastric balloon systems for potential complications and to instruct patients about the symptoms of potentially life-threatening complications [20]. In comparison to Genco et al. [21], our group of patients showed a higher complication rate of 21.4% (2× removal due to symptoms, 1× loss of a gastric balloon via naturalis), but the number of our study group was very small (*n* = 14 vs. *n* = 2,515 [21]). Finally, severe complications after the gastric balloon insertion seemed to be rare.

After gastric balloon insertion, we could show a reduction of structural fat, visceral fat, and subcutaneous fatty tissue (see Fig. 1a-c). There was also a significant reduction of the volume of the left liver lobe (see Fig. 3A). We could only find a study by Takihata et al. [22] investigating the effects of a gastric balloon therapy and an intensive lifestyle change including effects on structural fat compartments. Comparative studies have already qualitatively and quantitatively shown that dietary and sports-based measures have a different effect on visceral and subcutaneous fat than a gastric balloon therapy or bariatric surgery [23, 24]. The individual fatty tissue compartments also show clear structural and functional differences [25] and do not serve for only storing energy. The endocrine function of the fatty tissue shows hyperactivity in adiposity, which significantly contributes to negative health effects [26]. Especially, the reduction of visceral fat seems to be important, due to its negative effect on the cardiovascular risk profile [27, 28, 29]. An evaluation of the visceral and subcutaneous abdominal fat tissue after weight reduction is in the focus of interest [30]. Corresponding to the literature after weight reduction, [14] a reduction of subcutaneous and intra-abdominal fatty tissue must be assumed after the gastric balloon therapy. Unfortunately, we could not find data on the influence of gastric balloon therapy on all three abdominal fatty compartments in the same patient in the literature. Sekino et al. [31] described in 8 patients (median BMI 44.0 kg/m^2^) a reduction of the liver volume but no changes of the intra-abdominal visceral fat area, following the definition of the Japanese Society of Internal Medicine. The CT-based method may be useful for metabolic questions. We decided to measure the fatty tissue thickness between the aorta and corpus pancreatis, representing visceral fat in MRI. This area is anatomically well defined and insensitive against respiratory and digestive organ movement. According to our data, a loss of visceral and subcutaneous fat was described by Cabral in 25 patients via ultrasound [32]. We could not find a measurement of retroperitoneal fat in the literature during gastric balloon therapy.

Furthermore, a high proportion of visceral fat and a large left liver lobe hinder and harbor risks for a bariatric surgery. Frutos et al. [33] could show a reduction of the liver volume after gastric balloon therapy prior to laparoscopic gastric bypass. Our data of the reduction of the left liver volume (see Table 3A) are in concise with these data [33], the data of Sekino et al. [31] after gastric balloon treatment and the data of Vogt [14] after conservative weight reduction.

The m. psoas is known as sentinel muscle for the MRI-supported objectification of muscle mass changes [14, 15]. In our study, the left m. psoas shows a significant and, on the right side, no significant reduction of the cross-sectional area (see Fig. 2a, b). Thus, suggesting a loss of muscle mass. This is an undesirable effect, as sarcopenic adiposity in particular is predictably harmful [16]. However, loss of muscle mass has also been noticed following bariatric surgeries [34] and dietary measures [14, 35]. Physical activity combined with a diet rich in protein can prevent or minimize muscle loss [36]. In retrospect, it is difficult to discern whether this is caused by a protein-deficient diet or physical inactivity.

The gastric balloon's fill volume showed a largely stable behavior with insignificant and incomprehensible kinetics of the partially contained gas bubbles (Fig. 4A). We can see no overfilling of balloons, as discussed in several FDA letters [17, 20] and a review by Tate et al. [37], but changing volumes of gas bubbles in the balloons (Fig. 4A, B). No additional studies can be found regarding this observation. A positive or negative effect on the therapy is currently still to be determined.

### Limitations

Main limitations of the study are the small number (*n* = 12) of patients included and the wide deviation in the initial BMI (between 39.1 and 67 kg/m^2^). The small number of patients and the deviation in the initial BMI are caused by the limited indications for gastric balloon placement. Since the data were collected in an intraindividual paired setting (after insertion and before removal in the same patient), a statistical analysis seems applicable and conclusive. Consequently, the results were shown paired in the figures. Another limitation is the prolonged balloon insertion time of 25–43 weeks (30.1 ± 1.6 weeks, mean ± SEM), while manufacturer's instructions for safety are for 6 months (24 weeks). We tried to remove the gastric balloons after 6 months, which was not always possible.

## Conclusion

Although the gastric balloon is no longer recommended on a regular basis in the current German AWMF guidelines [4], it is a proven and relatively safe method for a controlled weight reduction if the patient does not desire surgery or surgery is not feasible for other reasons. The reduction of all abdominal body fat compartments is a finding yet to be published. The proven and undesired loss of muscle mass should be prevented with a high-protein diet and regular physical activity. The verified reductive effects on the left lobe of the liver and visceral fat are also particularly desirable in the preparation for bariatric surgery. The volume of the gastric balloon seems to be mainly stable during therapy, but changes are possible. In conclusion, these results open up interesting and comprehensive research approaches, thus also facilitating a positive reassessment of the gastric balloon within the context of adiposity therapy.

## Statement of Ethics

All procedures performed were in accordance with the ethical standards of the Institutional Research Committee and with the 1964 Helsinki Declaration and its later amendments or comparable ethical standards. The study was approved by the local University Medical Center Ethics Commission (Ethikkommission an der Universitätsmedizin Greifswald, Institut für Pharmakologie, Felix-Hausdorf-Straße 3, 17487 Greifswald, Germany) under the registration number BB24/12. Written informed consent was obtained from all individual participants included in the study.

## Conflict of Interest Statement

The authors have no conflicts of interest to declare.

## Funding Sources

This research did not receive grants from any funding agency in the public, commercial, or not-for-profit sectors.

## Author Contributions

Rebecca Keßler acquired and analyzed the data, drafted the work, and revised the work critically. Anne Glitsch designed the study, acquired the data, and revised the work critically. Björn Hübner analyzed and interpreted the data and wrote the manuscript. Simone Gärtner acquired and interpreted the data and revised the work critically. Antje Steveling and Maciej Patrzyk designed the study, interpreted the data, and revised the work critically. Wolfram Keßler designed the study; acquired, analyzed, and interpreted the data; and wrote the manuscript.

## Data Availability Statement

All data generated or analyzed during this study are included in this article. Further inquiries can be directed to the corresponding author.

## Notes

Rebecca Keßler and Anne Glitsch equally contributed to this research paper and share first authorship.
